# 4-[(1-Adamant­yl)carbamo­yl]pyridinium chloride

**DOI:** 10.1107/S1600536810052499

**Published:** 2010-12-24

**Authors:** Yingchun Wang

**Affiliations:** a Ordered Matter Science Research Center, Southeast University, Nanjing 210096, People’s Republic of China

## Abstract

In the title compound, C_16_H_21_N_2_O^+^·Cl^−^, the amide group makes a dihedral angle of 25.9 (1)° with respect to the pyridine ring. In the crystal, inter­molecular N—H⋯Cl bonds and weak C—H⋯Cl and C—H⋯O contacts link the cations and the anions into layers parallel to the *ac* plane. The layers are packed along [010] by hydro­phobic inter­actions between adamantane units.

## Related literature

For biomedical properties of adamantane-1-amine derivatives, see: Lees (2005 ▶); Nayyar *et al.* (2007 ▶). For ferroelectric properties of pyridinium salts, see: Ye *et al.* (2010 ▶); Zhang *et al.* (2010 ▶).
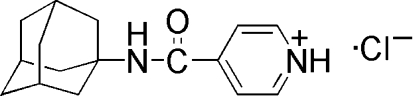


## Experimental

### 

#### Crystal data

C_16_H_21_N_2_O^+^·Cl^−^
*M* *_r_* = 292.80
Monoclinic, 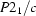

*a* = 7.117 (4) Å
*b* = 23.093 (13) Å
*c* = 11.241 (5) Å
β = 127.56 (2)°
*V* = 1464.5 (13) Å^3^
*Z* = 4
Mo *K*α radiation
μ = 0.26 mm^−1^
*T* = 293 K
0.20 × 0.20 × 0.20 mm

#### Data collection

Rigaku SCXmini diffractometer
Absorption correction: multi-scan (*CrystalClear*; Rigaku, 2005 ▶) *T*_min_ = 0.950, *T*_max_ = 0.950
14193 measured reflections
3377 independent reflections
2910 reflections with *I* > 2σ(*I*)
*R*_int_ = 0.042

#### Refinement

*R*[*F* ^2^ > 2σ(*F* ^2^)] = 0.056
*wR*(*F* ^2^) = 0.139
*S* = 1.11
3377 reflections
202 parameters
H-atom parameters constrained
Δρ_max_ = 0.23 e Å^−3^
Δρ_min_ = −0.22 e Å^−3^

### 

Data collection: *CrystalClear* (Rigaku, 2005 ▶); cell refinement: *CrystalClear*; data reduction: *CrystalClear*; program(s) used to solve structure: *SHELXS97* (Sheldrick, 2008 ▶); program(s) used to refine structure: *SHELXL97* (Sheldrick, 2008 ▶); molecular graphics: *SHELXTL/PC* (Sheldrick, 2008 ▶); software used to prepare material for publication: *SHELXTL/PC*.

## Supplementary Material

Crystal structure: contains datablocks I, global. DOI: 10.1107/S1600536810052499/si2302sup1.cif

Structure factors: contains datablocks I. DOI: 10.1107/S1600536810052499/si2302Isup2.hkl

Additional supplementary materials:  crystallographic information; 3D view; checkCIF report

## Figures and Tables

**Table 1:** Hydrogen-bond geometry (Å, °)

*D*—H⋯*A*	*D*—H	H⋯*A*	*D*⋯*A*	*D*—H⋯*A*
N1—H1*A*⋯Cl1^i^	0.90	2.16	3.017 (2)	160
N2—H2*A*⋯Cl1^ii^	0.90	2.50	3.293 (2)	147
C2—H2*B*⋯Cl1^iii^	0.96	2.79	3.535 (3)	136
C3—H3*A*⋯Cl1^iv^	0.96	2.78	3.536 (3)	136
C4—H4*A*⋯O1^ii^	0.96	2.35	3.203 (3)	147

Symmetry codes: (i) 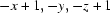; (ii) 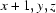; (iii) 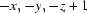; (iv) 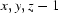.

**Fig. 1.**
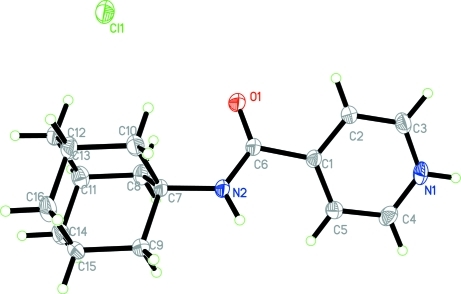
The molecular structure of the title compound, with the atomic numbering scheme. Displacement ellipsoids are drawn at the 30% probability level.

**Fig. 2.**
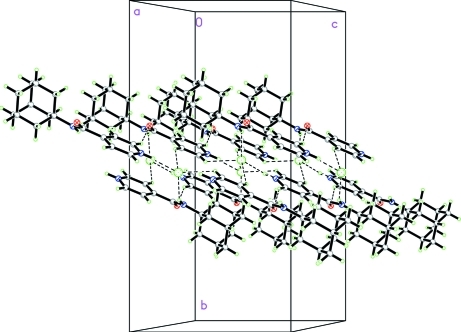
A view of a packing section of the title compound, stacking along the b axis. Dashed lines indicate hydrogen bonds.

## supplementary crystallographic information

### Comment

The study of amantadine and its derivatives has attracted much attention owing to their multifunction and technological applications in many areas, such as biomedicine (Lees 2005; Nayyar *et al.*2007). Amantadine can crystallize in different space groups owing to its randomness. As one part of our systematic research on dielectric, ferroelectric, and phase-transition materials (Ye *et al.* 2010; Zhang *et al.* 2010), we synthesize the title compound and investigated its dielectric property. In the range of 110 K to its melting point (428–432 K), the dielectric constant increases smoothly as a function of temperature. It means that this compound might not undergo a distinct structural phase transition in the measured temperature range.

The asymmetric unit of the title compound contains one protonated *N*- (1-adamantyl)isonicotinamide basic ion and one negative chlorine ion (Fig. 1). The torsion angles of C2—C1—C6—O1 and C2—C1—C6—N2 are 24.5 (3) ånd -157.5 (2) °, C5—C1—C6—O1 and C5—C1—C6—N2 are -151.3 (2) ° and 26.7 (3) °. Intermolecular N—H···Cl bonds and weak C—H···Cl and C—H···O contacts link cationic molecules parallel to (1 0 1) (Table 1). The layers are packed by hydrophobic interactions between adamantane units along the *b*-axis (Fig 2).

### Experimental

Isonicotinic acid 5 g was added in thionyl chloride (50 ml), and the mixture reacted at 353 K for 5 h. Then the solvate was removed under reduced pressure, the isonicotinoyl chloride was obtained. The l-aminodiamantane hydrochloride (10 mmol) and triethylamine 2.02 g (20 mmol) dissolved in chloroform (40 ml) at 273 K, then the isonicotinoyl chloride 1.51 g (10 mmol) was added. Then the reactant mixture was stired for 7 h at room temperature and some flaxen solid appeared. After filtering the mixture, the solid was dissolved in water and was neutralized with sodium carbonate, The mixed solution was extracted by dichloromethane. The *N*-(1-adamantyl)isonicotinamide was obtained when the dichloromethane was evaporated under reduced pressure.

The *N*(1-adamantyl)isonicotinamide 2.56 g (10 mmol) was dissolved in methanol and the chlorhydric acid 1 ml (12 mmol/ml) was added. The crystals suitable for structure determination were grown by slow evaporation of the filter solution at room temperature.

### Refinement

Positional parameters of all H atoms were calculated geometrically and were allowed to ride on the C and N atoms to which they are bonded, with N–H and C–H distances 0.90 Å and 0.96 Å, respectively. The isotropic displace ment parameters of the H atoms were refined freely with *U*_iso_(H) = 1.7*U*_eq_(N), and the *U*_iso_(H) at carbon atoms range between 1.1 and 1.6*U*_eq_(C).

### Crystal data

**Table:**

C_16_H_21_N_2_O^+^·Cl^−^	*F*(000) = 624
*M**_r_* = 292.80	*D*_x_ = 1.328 Mg m^−^^3^
Monoclinic, *P*2_1_/*c*	Mo *K*α radiation, λ = 0.71073 Å
Hall symbol: -P 2ybc	Cell parameters from 3642 reflections
*a* = 7.117 (4) Å	θ = 2.9–27.6°
*b* = 23.093 (13) Å	µ = 0.26 mm^−^^1^
*c* = 11.241 (5) Å	*T* = 293 K
β = 127.56 (2)°	Prism, colourless
*V* = 1464.5 (13) Å^3^	0.20 × 0.20 × 0.20 mm
*Z* = 4	

### Data collection

**Table:**

Rigaku SCXmini diffractometer	3377 independent reflections
Radiation source: fine-focus sealed tube	2910 reflections with *I* > 2σ(*I*)
graphite	*R*_int_ = 0.042
Detector resolution: 13.6612 pixels mm^-1^	θ_max_ = 27.6°, θ_min_ = 2.9°
ω scans	*h* = −9→9
Absorption correction: multi-scan (*CrystalClear*; Rigaku, 2005)	*k* = −30→30
*T*_min_ = 0.950, *T*_max_ = 0.950	*l* = −14→14
14193 measured reflections	

### Refinement

**Table:**

Refinement on *F*^2^	Primary atom site location: structure-invariant direct methods
Least-squares matrix: full	Secondary atom site location: difference Fourier map
*R*[*F*^2^ > 2σ(*F*^2^)] = 0.056	Hydrogen site location: inferred from neighbouring sites
*wR*(*F*^2^) = 0.139	H-atom parameters constrained
*S* = 1.11	*w* = 1/[σ^2^(*F*_o_^2^) + (0.0598*P*)^2^ + 0.5955*P*] where *P* = (*F*_o_^2^ + 2*F*_c_^2^)/3
3377 reflections	(Δ/σ)_max_ = 0.041
202 parameters	Δρ_max_ = 0.23 e Å^−^^3^
0 restraints	Δρ_min_ = −0.22 e Å^−^^3^

### Special details

**Table:**

Geometry. All e.s.d.'s (except the e.s.d. in the dihedral angle between two l.s. planes) are estimated using the full covariance matrix. The cell e.s.d.'s are taken into account individually in the estimation of e.s.d.'s in distances, angles and torsion angles; correlations between e.s.d.'s in cell parameters are only used when they are defined by crystal symmetry. An approximate (isotropic) treatment of cell e.s.d.'s is used for estimating e.s.d.'s involving l.s. planes.
Refinement. Refinement of *F*^2^ against ALL reflections. The weighted *R*-factor *wR* and goodness of fit *S* are based on *F*^2^, conventional *R*-factors *R* are based on *F*, with *F* set to zero for negative *F*^2^. The threshold expression of *F*^2^ > σ(*F*^2^) is used only for calculating *R*-factors(gt) *etc*. and is not relevant to the choice of reflections for refinement. *R*-factors based on *F*^2^ are statistically about twice as large as those based on *F*, and *R*- factors based on ALL data will be even larger.

### Fractional atomic coordinates and isotropic or equivalent isotropic displacement parameters (Å^2^)

**Table:**

	*x*	*y*	*z*	*U*_iso_*/*U*_eq_	
Cl1	0.13642 (10)	−0.01867 (2)	0.85883 (6)	0.03808 (17)	
O1	0.3301 (3)	−0.12489 (9)	0.51605 (18)	0.0531 (5)	
N1	0.6627 (4)	−0.03832 (8)	0.2831 (2)	0.0406 (5)	
H1A	0.6865	−0.0228	0.2198	0.070 (9)*	
N2	0.7203 (3)	−0.11813 (8)	0.71977 (19)	0.0345 (4)	
H2A	0.8612	−0.1036	0.7512	0.059 (8)*	
C1	0.5926 (4)	−0.08374 (9)	0.4772 (2)	0.0307 (4)	
C2	0.4096 (4)	−0.05633 (10)	0.3459 (2)	0.0375 (5)	
H2B	0.2555	−0.0532	0.3221	0.050 (7)*	
C3	0.4491 (4)	−0.03368 (10)	0.2503 (3)	0.0412 (5)	
H3A	0.3237	−0.0144	0.1599	0.053 (8)*	
C4	0.8423 (4)	−0.06503 (10)	0.4063 (3)	0.0426 (5)	
H4A	0.9931	−0.0680	0.4254	0.053 (8)*	
C5	0.8118 (4)	−0.08820 (10)	0.5065 (3)	0.0384 (5)	
H5A	0.9410	−0.1072	0.5959	0.050 (7)*	
C6	0.5359 (4)	−0.11061 (10)	0.5753 (2)	0.0349 (5)	
C7	0.7100 (3)	−0.14894 (8)	0.8312 (2)	0.0286 (4)	
C8	0.6137 (4)	−0.21041 (9)	0.7764 (2)	0.0381 (5)	
H8A	0.4549	−0.2086	0.6847	0.043 (7)*	
H8B	0.7099	−0.2307	0.7568	0.059 (8)*	
C9	0.9648 (4)	−0.15297 (10)	0.9762 (2)	0.0404 (5)	
H9A	1.0617	−0.1731	0.9569	0.057 (8)*	
H9B	1.0284	−0.1147	1.0111	0.044 (7)*	
C10	0.5588 (4)	−0.11689 (9)	0.8641 (3)	0.0376 (5)	
H10A	0.6192	−0.0784	0.8989	0.050 (7)*	
H10B	0.3989	−0.1139	0.7738	0.046 (7)*	
C11	0.6166 (4)	−0.24278 (9)	0.8968 (3)	0.0415 (5)	
H11A	0.5551	−0.2811	0.8615	0.057 (8)*	
C12	0.4621 (4)	−0.21057 (10)	0.9256 (3)	0.0427 (5)	
H12A	0.3033	−0.2080	0.8341	0.060 (8)*	
H12B	0.4575	−0.2313	0.9978	0.060 (8)*	
C13	0.5605 (4)	−0.15005 (10)	0.9832 (3)	0.0403 (5)	
H13A	0.4650	−0.1300	1.0037	0.064 (8)*	
C14	0.8703 (5)	−0.24643 (10)	1.0405 (3)	0.0463 (6)	
H14A	0.8734	−0.2675	1.1153	0.056 (8)*	
H14B	0.9668	−0.2668	1.0211	0.067 (9)*	
C15	0.9674 (4)	−0.18568 (11)	1.0960 (2)	0.0419 (5)	
H15A	1.1271	−0.1879	1.1867	0.053 (7)*	
C16	0.8150 (5)	−0.15348 (11)	1.1268 (3)	0.0450 (6)	
H16A	0.8771	−0.1152	1.1629	0.050 (7)*	
H16B	0.8180	−0.1735	1.2028	0.059 (8)*	

### Atomic displacement parameters (Å^2^)

**Table:**

	*U*^11^	*U*^22^	*U*^33^	*U*^12^	*U*^13^	*U*^23^
Cl1	0.0399 (3)	0.0436 (3)	0.0357 (3)	−0.0034 (2)	0.0256 (3)	0.0040 (2)
O1	0.0297 (9)	0.0891 (13)	0.0340 (9)	−0.0059 (8)	0.0160 (7)	0.0178 (8)
N1	0.0554 (12)	0.0444 (10)	0.0346 (10)	−0.0066 (9)	0.0340 (10)	0.0014 (8)
N2	0.0293 (9)	0.0497 (10)	0.0246 (8)	−0.0055 (7)	0.0166 (8)	0.0054 (7)
C1	0.0320 (10)	0.0365 (10)	0.0262 (10)	−0.0031 (8)	0.0189 (9)	0.0004 (8)
C2	0.0336 (11)	0.0460 (12)	0.0328 (11)	0.0025 (9)	0.0203 (10)	0.0092 (9)
C3	0.0462 (13)	0.0443 (12)	0.0306 (11)	−0.0007 (10)	0.0220 (11)	0.0076 (9)
C4	0.0419 (13)	0.0553 (14)	0.0440 (13)	−0.0019 (10)	0.0331 (12)	−0.0004 (10)
C5	0.0339 (11)	0.0497 (13)	0.0333 (11)	0.0041 (9)	0.0213 (10)	0.0064 (9)
C6	0.0307 (11)	0.0459 (12)	0.0296 (11)	−0.0006 (8)	0.0191 (9)	0.0068 (8)
C7	0.0291 (10)	0.0354 (10)	0.0229 (9)	−0.0015 (8)	0.0166 (8)	0.0036 (7)
C8	0.0458 (13)	0.0383 (12)	0.0332 (11)	−0.0039 (9)	0.0257 (11)	−0.0036 (9)
C9	0.0295 (11)	0.0546 (14)	0.0319 (11)	−0.0049 (9)	0.0160 (10)	0.0079 (10)
C10	0.0453 (13)	0.0372 (11)	0.0369 (12)	0.0088 (9)	0.0286 (11)	0.0078 (9)
C11	0.0527 (14)	0.0303 (11)	0.0400 (12)	−0.0070 (9)	0.0274 (11)	0.0006 (9)
C12	0.0372 (12)	0.0569 (14)	0.0361 (12)	−0.0047 (10)	0.0233 (11)	0.0091 (10)
C13	0.0492 (14)	0.0478 (13)	0.0382 (12)	0.0093 (10)	0.0340 (12)	0.0066 (9)
C14	0.0523 (15)	0.0427 (13)	0.0482 (14)	0.0117 (10)	0.0328 (13)	0.0168 (10)
C15	0.0294 (11)	0.0583 (14)	0.0267 (11)	−0.0015 (9)	0.0112 (9)	0.0122 (9)
C16	0.0571 (15)	0.0491 (14)	0.0297 (12)	−0.0087 (11)	0.0270 (12)	−0.0011 (9)

### Geometric parameters (Å, °)

**Table:**

O1—C6	1.228 (3)	C9—C15	1.535 (3)
N1—C3	1.332 (3)	C9—H9A	0.9600
N1—C4	1.332 (3)	C9—H9B	0.9601
N1—H1A	0.9000	C10—C13	1.536 (3)
N2—C6	1.339 (3)	C10—H10A	0.9602
N2—C7	1.480 (2)	C10—H10B	0.9599
N2—H2A	0.9000	C11—C12	1.517 (3)
C1—C5	1.388 (3)	C11—C14	1.523 (4)
C1—C2	1.390 (3)	C11—H11A	0.9599
C1—C6	1.519 (3)	C12—C13	1.520 (3)
C2—C3	1.369 (3)	C12—H12A	0.9599
C2—H2B	0.9601	C12—H12B	0.9600
C3—H3A	0.9599	C13—C16	1.526 (3)
C4—C5	1.379 (3)	C13—H13A	0.9601
C4—H4A	0.9601	C14—C15	1.520 (4)
C5—H5A	0.9599	C14—H14A	0.9601
C7—C10	1.526 (3)	C14—H14B	0.9600
C7—C8	1.533 (3)	C15—C16	1.518 (3)
C7—C9	1.533 (3)	C15—H15A	0.9600
C8—C11	1.535 (3)	C16—H16A	0.9599
C8—H8A	0.9600	C16—H16B	0.9599
C8—H8B	0.9600		
			
C3—N1—C4	122.33 (19)	C7—C10—C13	109.73 (17)
C3—N1—H1A	118.9	C7—C10—H10A	109.9
C4—N1—H1A	118.8	C13—C10—H10A	110.0
C6—N2—C7	124.82 (18)	C7—C10—H10B	109.6
C6—N2—H2A	117.5	C13—C10—H10B	109.4
C7—N2—H2A	117.7	H10A—C10—H10B	108.2
C5—C1—C2	118.42 (19)	C12—C11—C14	110.2 (2)
C5—C1—C6	123.72 (18)	C12—C11—C8	109.11 (19)
C2—C1—C6	117.73 (19)	C14—C11—C8	109.4 (2)
C3—C2—C1	120.0 (2)	C12—C11—H11A	109.5
C3—C2—H2B	119.9	C14—C11—H11A	109.5
C1—C2—H2B	120.1	C8—C11—H11A	109.1
N1—C3—C2	119.8 (2)	C11—C12—C13	109.63 (18)
N1—C3—H3A	119.8	C11—C12—H12A	109.6
C2—C3—H3A	120.3	C13—C12—H12A	109.5
N1—C4—C5	120.0 (2)	C11—C12—H12B	109.8
N1—C4—H4A	119.9	C13—C12—H12B	110.1
C5—C4—H4A	120.1	H12A—C12—H12B	108.2
C4—C5—C1	119.4 (2)	C12—C13—C16	110.15 (19)
C4—C5—H5A	120.3	C12—C13—C10	109.30 (19)
C1—C5—H5A	120.2	C16—C13—C10	108.74 (19)
O1—C6—N2	125.90 (19)	C12—C13—H13A	109.7
O1—C6—C1	118.23 (19)	C16—C13—H13A	109.2
N2—C6—C1	115.83 (18)	C10—C13—H13A	109.8
N2—C7—C10	112.20 (17)	C15—C14—C11	109.43 (18)
N2—C7—C8	110.26 (16)	C15—C14—H14A	110.2
C10—C7—C8	109.73 (17)	C11—C14—H14A	109.7
N2—C7—C9	107.00 (16)	C15—C14—H14B	109.6
C10—C7—C9	108.87 (18)	C11—C14—H14B	109.7
C8—C7—C9	108.68 (17)	H14A—C14—H14B	108.2
C11—C8—C7	109.54 (17)	C16—C15—C14	109.7 (2)
C11—C8—H8A	110.0	C16—C15—C9	109.31 (19)
C7—C8—H8A	109.7	C14—C15—C9	109.5 (2)
C11—C8—H8B	109.7	C16—C15—H15A	109.4
C7—C8—H8B	109.7	C14—C15—H15A	109.5
H8A—C8—H8B	108.2	C9—C15—H15A	109.4
C15—C9—C7	109.69 (17)	C15—C16—C13	109.68 (19)
C15—C9—H9A	109.6	C15—C16—H16A	109.8
C7—C9—H9A	109.6	C13—C16—H16A	110.1
C15—C9—H9B	110.1	C15—C16—H16B	109.6
C7—C9—H9B	109.6	C13—C16—H16B	109.5
H9A—C9—H9B	108.2	H16A—C16—H16B	108.1
			
C5—C1—C2—C3	−1.1 (3)	C8—C7—C9—C15	−59.8 (2)
C6—C1—C2—C3	−177.1 (2)	N2—C7—C10—C13	−178.48 (17)
C4—N1—C3—C2	0.7 (3)	C8—C7—C10—C13	58.6 (2)
C1—C2—C3—N1	0.4 (3)	C9—C7—C10—C13	−60.2 (2)
C3—N1—C4—C5	−1.1 (4)	C7—C8—C11—C12	60.1 (2)
N1—C4—C5—C1	0.4 (4)	C7—C8—C11—C14	−60.6 (2)
C2—C1—C5—C4	0.6 (3)	C14—C11—C12—C13	58.8 (2)
C6—C1—C5—C4	176.4 (2)	C8—C11—C12—C13	−61.3 (2)
C7—N2—C6—O1	4.6 (4)	C11—C12—C13—C16	−58.4 (2)
C7—N2—C6—C1	−173.26 (18)	C11—C12—C13—C10	61.0 (2)
C5—C1—C6—O1	−151.3 (2)	C7—C10—C13—C12	−59.5 (2)
C2—C1—C6—O1	24.5 (3)	C7—C10—C13—C16	60.8 (2)
C5—C1—C6—N2	26.7 (3)	C12—C11—C14—C15	−59.6 (2)
C2—C1—C6—N2	−157.5 (2)	C8—C11—C14—C15	60.4 (3)
C6—N2—C7—C10	−66.8 (3)	C11—C14—C15—C16	59.8 (2)
C6—N2—C7—C8	55.8 (3)	C11—C14—C15—C9	−60.2 (3)
C6—N2—C7—C9	173.9 (2)	C7—C9—C15—C16	−59.9 (2)
N2—C7—C8—C11	177.02 (18)	C7—C9—C15—C14	60.2 (3)
C10—C7—C8—C11	−58.9 (2)	C14—C15—C16—C13	−59.6 (2)
C9—C7—C8—C11	60.0 (2)	C9—C15—C16—C13	60.6 (2)
N2—C7—C9—C15	−178.89 (18)	C12—C13—C16—C15	59.0 (2)
C10—C7—C9—C15	59.6 (2)	C10—C13—C16—C15	−60.8 (2)

### Hydrogen-bond geometry (Å, °)

**Table:**

*D*—H···*A*	*D*—H	H···*A*	*D*···*A*	*D*—H···*A*
N1—H1A···Cl1^i^	0.90	2.16	3.017 (2)	160.
N2—H2A···Cl1^ii^	0.90	2.50	3.293 (2)	147.
C2—H2B···Cl1^iii^	0.96	2.79	3.535 (3)	136.
C3—H3A···Cl1^iv^	0.96	2.78	3.536 (3)	136.
C4—H4A···O1^ii^	0.96	2.35	3.203 (3)	147.

Symmetry codes: (i) −*x*+1, −*y*, −*z*+1; (ii) *x*+1, *y*, *z*; (iii) −*x*, −*y*, −*z*+1; (iv) *x*, *y*, *z*−1.
